# Retraction: Transcriptional Repressor Gfi1 Integrates Cytokine-Receptor Signals Controlling B-Cell Differentiation

**DOI:** 10.1371/annotation/4f75f09f-a8f0-4cae-b75f-30498d6b44d4

**Published:** 2012-07-26

**Authors:** Chozhavendan Rathinam, Christoph Klein

The authors wish to retract the article "Transcriptional repressor

Gfi1 integrates cytokine-receptor signals controlling B-cell differentiation" by Chozhavendan Rathinam and Christoph Klein. PLoS ONE 2007, 2(3):e306.

After publication of the article, concerns were raised regarding the control bands in Figure 5A, and in particular, whether the bands represent total STAT5. Examination of the original Western blot data revealed that primary Western blot documents were not archived with due diligence. As a consequence, the doubts about the proper representation of the control bands in Figure 5A could not be unambiguously resolved. This course of action is in line with the recommendation issued by Hannover Medical School.

